# Neoantigen cancer vaccine augments anti-CTLA-4 efficacy

**DOI:** 10.1038/s41541-022-00433-9

**Published:** 2022-02-02

**Authors:** Erika Salvatori, Lucia Lione, Mirco Compagnone, Eleonora Pinto, Antonella Conforti, Gennaro Ciliberto, Luigi Aurisicchio, Fabio Palombo

**Affiliations:** 1 Takis, Rome, Italy; 2 Neomatrix, Rome, Italy; 3 Evvivax, Rome, Italy; 4 Scientific Directorate, IRCCS Regina Elena National Cancer Institute, Rome, Italy

**Keywords:** Applied immunology, Cancer

## Abstract

Immune checkpoint inhibitors (ICI) based on anti-CTLA-4 (αCTLA-4) and anti-PD1 (αPD1) are being tested in combination with different therapeutic approaches including other immunotherapies such as neoantigen cancer vaccines (NCV). Here we explored, in two cancer murine models, different therapeutic combinations of ICI with personalized DNA vaccines expressing neoantigens and delivered by electroporation (EP). Anti-cancer efficacy was evaluated using vaccines with or without CD4 epitopes. Therapeutic DNA vaccines showed synergistic effects in different therapeutic protocols including established large tumors. Flow cytometry (FC) was utilized to measure CD8, CD4, Treg, and switched B cells as well as neoantigen-specific immune responses, which were also measured by IFN-γ ELIspot. Immune responses were augmented in combination with αCTLA4 but not with αPD1 in the MC38 tumor-bearing mice, significantly impacting tumor growth. Similarly, neoantigen-specific T cell immune responses were enhanced in combined treatment with αCTLA-4 in the CT26 tumor model where large tumors regressed in all mice, while monotherapy with αCTLA-4 was less efficacious. In line with previous evidence, we observed an increased switched B cells in the spleen of mice treated with αCTLA-4 alone or in combination with NCV. These results support the use of NCV delivered by DNA-EP with αCTLA-4 and suggest a new combined therapy for clinical testing.

## Introduction

Developing innovative combination protocols to boost the efficacy of immune checkpoint inhibitors (ICI) is one of the challenges in cancer immunotherapy. In the last decade, monoclonal antibodies targeting the CTLA-4 and PD1 pathways have been approved as anticancer drugs for the treatment of several cancer types^1^. More recently it was discovered that the therapeutic success of ICI is linked to the activation of an immune response against neoantigens^2^, which are tumor-specific mutations recognized by the host immune system^3^. Direct targeting of neoantigens with personalized cancer vaccines is appealing for many reasons, including the potential lack of immunological tolerance and intrinsic tumor specificity. Neoantigen cancer vaccine (NCV) technology has been explored in preclinical tumor models using various delivery systems, including peptides^4–10^, long peptides^11^, RNA^12,13^, DNA^14,15^, and nanoparticles^16^. However, promising results in preclinical animal models did not translate in clinical benefit as monotherapy or in combination with anti-PD1 (αPD1)^17^ despite the detection of strong immune responses in most NCV-treated patients^18–23^. The lack of correlation requires a more in-depth knowledge of the mechanisms of action of the targeted pathway. Indeed, anti-CTLA-4 (αCTLA-4) and αPD1 are supposed to act at different levels, with the first promoting T cell activation at lymph nodes and the latter reactivating T cells in the tumor^24^. This conclusion is backed up by the large therapeutic advantage of combining αCTLA-4 and αPD1 therapy, even though efficacy comes at the expenses of severe adverse effects^25^. One potential explanation may be the general activation of the immune response against self-antigens expressed by normal tissues, whereas the combination of ICI with the NCV may spare normal tissue while boosting cancer selectivity.

The efficacy of the ICI-vaccine combination may also be affected by the type of vaccine platform. Previous evidence has shown that vaccine formulation and the vaccination protocol can dictate synergy with αCTLA-4 and αPD1 therapy^26^. The αCTLA-4 role in priming T cell response was investigated with a glioblastoma cell lysate vaccine treated with an NK ligand. A predominant CD4^+^ response and a synergy with ICI were observed^27^. Moreover, external factors such as the microbiota can modulate the efficacy of a NCV expressed by a plasmid DNA and administered by electroporation (EP)^28^.

Most of the preclinical studies with NCV were conducted with two colon cancer models the MC38 and CT26 cells^5,9,12–15,29–35^ while the combination of NCV with ICI was explored mainly with αPD1^31^. Here we explored the impact of therapeutic NCV using different vaccination protocols in combination with ICI. Our results suggest that the DNA-EP delivery of NCV is more effective with αCTLA-4 rather than with αPD1 in terms of immunological and antitumor effects.

## Results

### DNA-EP vaccination enhances the antitumor effect of αCTLA-4 in two different colon cancer models

**Fig. 1:**
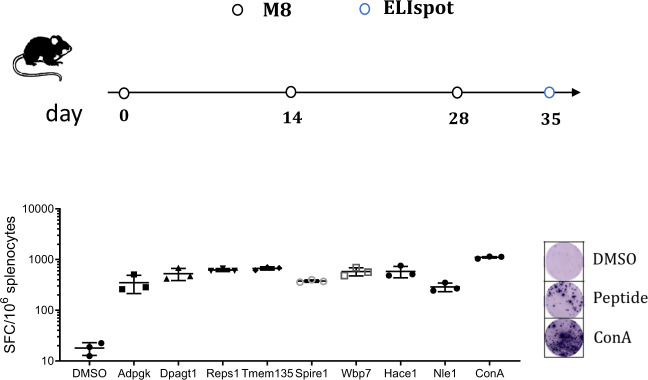
Immune responses induced by M8 vaccine. C57Bl/6 mice were vaccinated at day 0, 14 and 28, while neoantigen-specific immune responses were analyzed on day 35 on splenocytes by IFNγ ELISpot stimulated with individual peptides. ConA served as a positive control. Neoantigen-specific measurements are reported corresponding to the neoantigens listed in Table 1. Each symbol represents an individual sample (*n* = 3). A representative image of the experiment is on the right. Values are from one of two experiments.

The amount of immunogenic neoantigens is a vital component in preventing tumor progression, as we and others have established^12,15,31^. To expand on this concept, we generated M8, a DNA plasmid vaccine expressing eight MC38 neoantigens in the form of 28-mers (Table 1). Analysis of the neoantigen individual immune response elicited by M8 administration in C57Bl/6 mice was performed by IFNγ ELISpot, confirming vaccine immunogenicity (Fig. 1).

**Table 1:** M8 vaccine expressing MC38 neoantigens (see Fig. 1).

#	Symbol	WT	Neoantigen
1	Adpgk	GIPVHLELASMTN**R**ELMSSIVHQQVFPT	GIPVHLELASMTN**M**ELMSSIVHQQVFPT
2	Dpagt1	SLVISASIIVFNL**V**ELEGDYRDDHIFSL	SLVISASIIVFNL**L**ELEGDYRDDHIFSL
3	Reps1	GRVLELFRAAQL**P**NDVVLQIMELCGATR	GRVLELFRAAQL**A**NDVVLQIMELCGATR
4	Tmem135	LLRLTKGRFALMN**R**KALDVFGTGASREF	LLRLTKGRFALMN**L**KALDVFGTGASREF
5	Spire1	GEKRSISAIRSYQ**D**VMKICAAHLPTESE	GEKRSISAIRSYQ**Y**VMKICAAHLPTESE
6	Wbp7	LSSCLSNFHFMCA**R**ASYCIFQDDKKVFC	LSSCLSNFHFMCA**L**ASYCIFQDDKKVFC
7	Hace1	TELRMTRAIQPQI**N**AFLQGFHMFIPPSL	TELRMTRAIQPQI**Y**AFLQGFHMFIPPSL
8	Nle1	GHGHWVNTMALST**D**YALRTGAFEPAEAT	GHGHWVNTMALST**Y**YALRTGAFEPAEAT

Mutated amino acids are highlighted in bold and underlined.

**Fig. 2:**
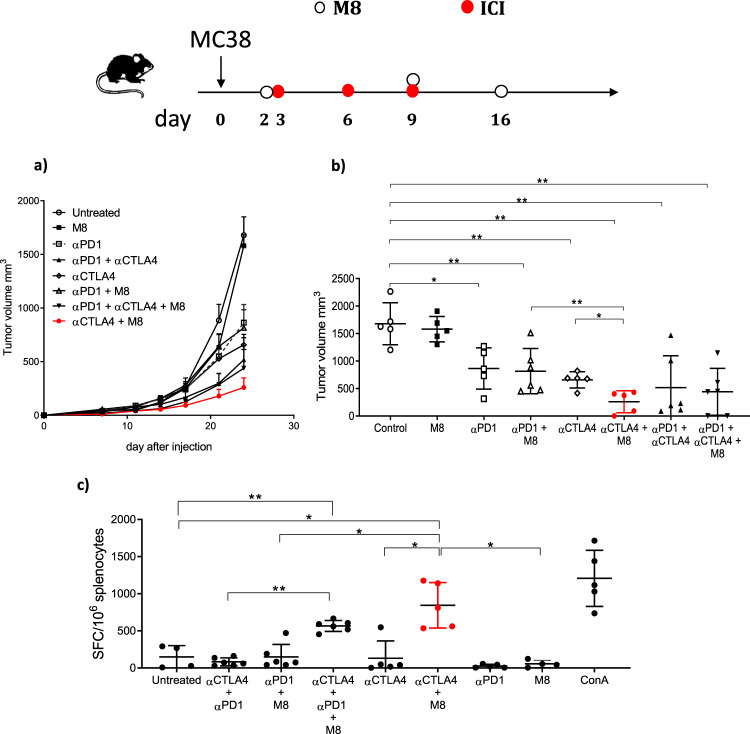
Therapeutic effect of M8 in combination with ICI. C57Bl/6 mice were inoculated s.c. with MC38 cells and treated with M8 vaccine delivered by EP and ICI according to the experimental scheme. **a** Tumor volumes followed over time. **b** Final tumor volume measurements, single tumor volumes are depicted from one experiment out of two performed. Mann–Whitney tests were conducted *p < 0,05 **p < 0.01 ***p < 0.001. **c** At day 27, mice were sacrificed and IFNγ producing cells were evaluated by IFNγ ELISpot assay with splenocytes restimulated with neoantigens pool or ConA as a positive control. Significance was determined using Mann–Whitney test, *p < 0,05 **p < 0.01 ***p < 0.001. Six animals per group were utilized. Each symbol represents an individual sample with the error bars representing the s.e.m. Only the most relevant statistics were reported.

We then asked whether the M8 vaccine could improve the antitumor efficacy when administered in combination with αPD1 and/or αCTLA-4. To this aim, C57Bl/6 mice were injected with MC38 cells and M8 administration started two days later in a therapeutic setting. As reported in Fig. 2A, B, the single αCTLA-4 or αPD1 and their combination significantly reduced tumor growth while M8 alone did not. Interestingly, the combined therapy of M8 with either αPD1 or αCTLA-4 was not equally effective.

The antitumor effects induced by the M8 vaccine combined with αPD1 were comparable with the αPD1 treatment alone, suggesting that the therapeutic effect was driven by the latter. Similarly, the dual treatment αCTLA-4/αPD1 did not improve over the single therapy. In contrast, the treatment with M8/αCTLA-4 resulted in a significant reduction of tumor growth with respect to αCTLA-4 alone, thus suggesting a possible role of the M8 vaccine in enhancing the antitumoral αCTLA-4 effect. To further support this observation, we evaluated the T cell response induced in the spleen at the time of sacrifices (Fig. 2C). Neoantigen-specific T cell response was significantly increased over the control mice only in mice treated with M8/αCTLA-4, which was even more effective than the triple treatment, M8/αCTLA-4/αPD1, although it did not reach statistical significance. This result suggests a synergistic effect of αCTLA-4 and M8 vaccine delivered by EP.

Recent evidence showed that the presence of a CD4 helper epitope may improve the antitumor response induced by NCV^35^. To verify this in the context of DNA-EP, we introduced helper CD4 epitopes at the C terminus of the M2 vaccine^15^ containing CD8 epitopes as nonamers, thus generating M2h vaccine. FC confirmed the immunogenicity of CD4 epitopes and showed an increase of neoantigen-specific CD8^+^IFNγ^+^ and CD8^+^TNFα^+^ (Supplementary Fig. 1A). This observation was further confirmed by IFNγ^+^ ELIspot assay (Supplementary Fig. 1B).

**Fig. 3:**
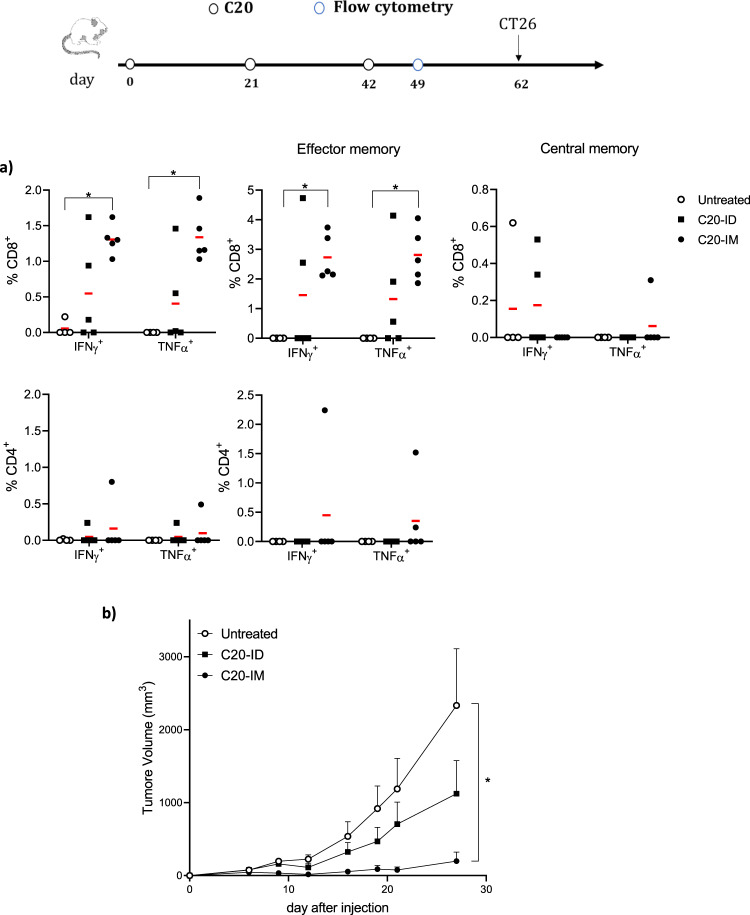
Prophylactic C20-IM induces a significant reduction in tumor growth. **a** Balb/c mice were vaccinated with the C20 vaccine at days 0, 21, and 42 and challenged with CT26 cells on day 62. One week after the last immunization (day 49), mice were bled retro-orbitally to monitor T cell immune response against CT26-neoepitopes by intracellular staining. Panel describes CD8 and CD4 neoantigen-specific effector and central memory T cell responses measured by FC using the gating strategy depicted in Supplementary Fig. 2. The stimulation pool included the 15 peptides listed in Table 2. **b** The panel depicts CT26 tumor growth overtime of one out of two experiments performed. Five animals per group were utilized. Each symbol represents an individual sample with the error bars representing the s.e.m. Significance was determined using Mann–Whitney test (*p < 0,05).

Previous evidence showed that CD4 epitopes could be relevant in the cotreatment of CT26 tumors with αCTLA-4^36^. Therefore, to combine CD4 and CD8 neoantigens with ICI in a second tumor model, we selected CD4 and CD8 neoantigens expressed in the CT26 colon cancer cells^37^ and we generated C20, a vaccine expressing twenty CT26 neoantigens in the form of 28-mers (Supplementary Table 1). In view of the potential role played by the site of vaccination^26^ and the evidence showing effective DNA vaccination with EP in other tissues^38^, we compared the therapeutic effect of C20 vaccine delivered intramuscularly (IM) vs. intradermally (ID). Neoantigen-specific immune response was analyzed by FC one week after last immunization showing significant CD8^+^IFNγ^+^ and CD8^+^TNFα^+^responses, which were also significantly elevated as effector memory CD8^+^IFNγ^+^, and CD8^+^TNFα^+^ in the IM protocol (C20-IM) with respect to control mice (Fig. 3a). Neoantigen-specific CD8^+^IFNγ^+^ and CD8^+^TNFα^+^ T cells were induced also in C20 delivered ID (C20-ID) but were not statistically significant. To evaluate the immune response against single neoantigens, splenocytes were analyzed. Table 2 summarizes the characterization of the immune responses; we observed two CD8 epitopes (Mtch1, Tmem87), two CD4 epitopes (Aldh1, Dhx35), and two CD8 and CD4 epitopes (E2f8, Slc20a1).

**Table 2:** Immunogenic neoantigens expressed by C20 vaccine.

					ICS	
Gene	28mer	15mer mutated	WT	IFNγ ELIspot Assay (SFC 1 × 10^6^ cells)	CD4 (%)	CD8 (%)
Aldh1 8a1	HSGQNHLKEMAI**S**VLEARACAAAGQ	QNHLKEMAI**S**VLEAR	QNHLKEMAI**P**VLEAR	141	0,055	–
		KEMAI**S**VLEARACAA	KEMAI**P**VLEARACAA	124,5	0,08	–
Dhx35	VIQTSKYYMRDV**I**AIESAWLLELAP	QTSKYYMRDV**I**AIES	QTSKYYMRDV**T**AIES	136	not tested	not tested
		YYMRDV**I**AIESAWLL	YYMRDV**T**AIESAWLL	176,5	0,04	–
E2f8	ILPQAPSGPSYA**T**YLQPAQAQMLTP	LPQAPSGPSYA**T**YLQ	LPQAPSGPSYA**I**YLQ	612	0,032	–
		PSGPSYA**T**YLQPAQA	PSGPSYA**I**YLQPAQA	594	–	0,045
		SYA**T**YLQPAQAQMLT	SYA**I**YLQPAQAQMLT	45,5	–	0,44
Mtch1	SWIHCWKYLSVQS**S**QLFRGSSLLFRR	HCWKYLSVQS**S**QLFR	HCWKYLSVQS**G**QLFR	822	–	0,24
		YLSVQS**S**QLFRGSSL	YLSVQS**G**QLFRGSSL	158	not tested	not tested
Slc20a1	KPLRRNNSYTSY**I**MAICGMPLDSFR	PLRRNNSYTSY**I**MAI	PLRRNNSYTSY**T**MAI		–	0,15
		NNSYTSY**I**MAICGMP	NNSYTSY**T**MAICGMP	193	0,06	–
		TSY**I**MAICGMPLDSF	TSY**T**MAICGMPLDSF	110	0,08	–
Tmem87	QAIVRGCSMPGPW**R**SGRLLVSRRWSVE	GCSMPGPW**R**SGRLLV	GCSMPGPW**G**SGRLLV	733,5	–	0,58
		PGPW**R**SGRLLVSRRW	PGPW**G**SGRLLVSRRW	457	not tested	not tested
Agxt2l2	EHIHRAGGLFVAD**A**IQVGFGRIGKHFW	HIHRAGGLFVAD**A**IQ	HIHRAGGLFVAD**E**IQ	47,5	–	–

Mutated amino acids are highlighted in bold and underlined.

In line with our previous report for the MC38 tumor model^15^, prophylactic C20-IM vaccination resulted in significant tumor delay (Fig. 3B). Reduced tumor growth was also observed in mice vaccinated ID, although it was not statistically significant. To verify whether a different inflammatory response was associated with the reduced antitumor efficacy observed in the C20-ID treated mice, circulating cytokines and chemokines were measured by Luminex multiparameter assay. Out of 14 analytics, only a limited induction of IL17A was observed in mice treated by ID administration (Supplementary Fig. 3A). Finally, we looked at the gene expression using the luciferase reporter gene in the ID vs. IM. A stronger level of luciferase was observed in the IM condition (Supplementary Fig. 2B). Further experiments with more efficient ID protocol and with a larger number of animals are required to explain the inefficient tumor protection in this prophylactic protocol.

**Fig. 4:**
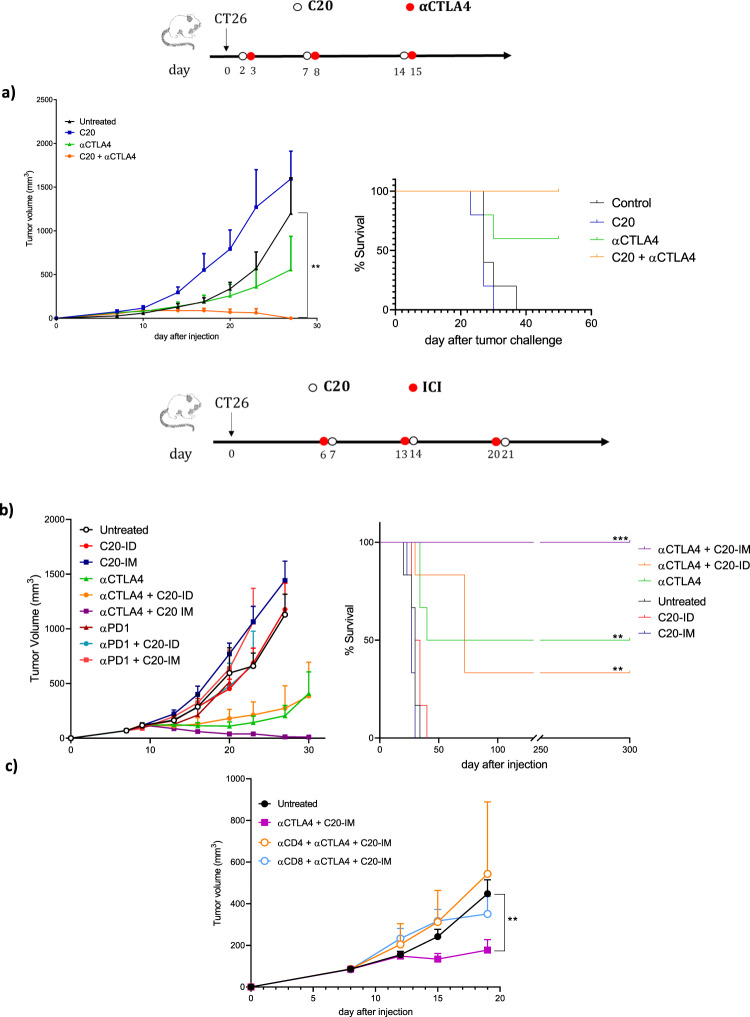
Therapeutic effect of C20 in combination with ICI in two different schedules of vaccination. **a** Balb/c mice were inoculated s.c. with CT26 cells and treated with C20 and ICI starting from day 2 as depicted in the experimental scheme. Tumor volume and survival curve. **b**, **c** Balb/c mice were inoculated s.c. with CT26 cells and treated with ICI and NCV according to the experimental scheme. **b** Tumor (50–100 mm^3^) bearing mice were randomized at day 6 and treated with αCTLA-4 and vaccinated with C20 the day after. The treatment was repeated weekly as described in the scheme. **b** Tumor volume measurements and survival curve. This experiment was conducted only once (**C**) CT26 tumor growth in CD4 or CD8 depleted mice treated as in panel (**b**). This experiment was repeated twice with similar results. Six animals per group were utilized with the error bars representing the s.e.m. Significance was determined using Mann–Whitney and Log-rank (Mantel–Cox) test **p < 0.01 ***p < 0.001.

Having observed an antitumor effect with the prophylactic C20-IM vaccine, we moved to a therapeutic setting also in this tumor model. To better reflect the longer interval used in the clinical schedule, ICI and the vaccine treatments were performed every five or seven days starting two days after tumor cell implantation. The therapeutic treatment with C20-IM followed by αCTLA-4 showed complete tumor regression in cotreated animals, while a partial regression was observed in the group of mice treated only with αCTLA-4 (Fig. 4A). We then investigated the combined treatment in established CT26 tumors with a slightly modified protocol. It has been shown that αCTLA-4 injection the day before a cell-based vaccine induced significant tumor regression of an orthotopic tumor model^27^. Therefore, mice bearing measurable tumors (50–100 mm^3^) were treated weekly with ICI and vaccinated the day after with the C20 vaccine for three weeks (Fig. 4B and Supplementary Fig. 4). Tumor stabilization was observed in mice treated with αCTLA-4, resulting in 50 % of survivors at the end of the experiment.

In contrast, tumor regression was observed in 100% of mice treated with αCTLA-4 and vaccinated with C20-IM (αCTLA-4/C20-IM). All mice in this group were tumor-free for more than 250 days. αCTLA-4 followed by C20-ID (αCTLA-4/C20-ID) resulted in a transient prolongation of survival, with some animals relapsing later. Notably, the monotherapy with C20 vaccine delivered either by ID or IM vaccination was ineffective as αPD1 alone or in combination with C20-IM or C20-ID vaccines. The therapeutic efficacy of αCTLA-4/C20-IM was confirmed in a second experiment up to day 70 when mice were challenged on the opposite flank with the CT26 tumor cells (Supplementary Fig. 4A). To verify whether a memory immune response was induced, survival mice were sacrificed at day 258 and memory immune response was analyzed in the splenocytes. A clear signal was observed in mice treated with αCTLA-4/C20-IM (Supplementary Fig. 5). In line with this evidence, mice treated with αCTLA-4/C20-IM were 100% protected in the rechallenge experiment (Supplementary Fig. 4B). To characterize the mechanism of action of αCTLA-4/C20-IM we performed T cell depletion. Both anti CD4 and anti CD8 abolished anti-tumor activity induced by αCTLA-4/C20-IM (Fig. 4C). These results extend to the CT26 tumor model the combination of NCV delivered by DNA-EP and αCTLA-4 and suggest that the protocol with ICI followed by NCV could be more effective.

### Neoantigen specific T and switched B cells correlate with tumor regression in mice treated with αCTLA-4 and C20-IM

**Fig. 5:**
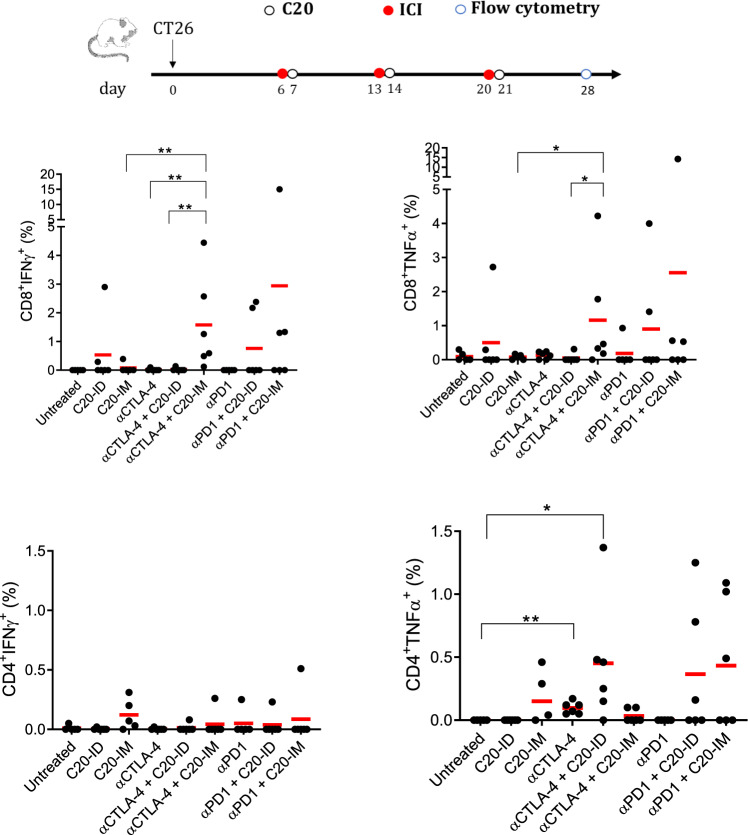
CD8^+^IFNγ^+^ response increased in αCTLA-4 + C20-IM treated mice. Tumor-bearing mice were treated as described in the experimental scheme and neoantigen-specific immune response was analyzed by FC in the peripheral blood one week after the last treatment (on day 28). Production of IFNγ and TNFα by CD4^+^ and CD8^+^ T cells was measured by FC using the gating strategy depicted in Supplementary Fig. 2 upon restimulation with the pool of neoantigen peptides listed in Table 2. This experiment was conducted twice with similar results. Each symbol represents an individual sample (*n* = 6). Significance was determined using Mann–Whitney test, *p < 0,05 **p < 0.01.

To identify immune correlates with the antitumor activity observed against established CT26 tumors, neoantigen-specific T cell immune responses were analyzed in peripheral blood by FC (Fig. 5). Tumor-bearing mice showed scattered immune responses. Treatment with αCTLA-4/C20-IM showed a significant neoantigen-specific CD8^+^IFNγ^+^ in all mice and CD8^+^TNFα^+^ T cell response in five out of six. C20-IM elicited a neoantigen-specific peripheral CD4^+^IFNγ^+^ in four out of six mice that were lessened to only one mouse in the cotreatment with αCTLA-4. In contrast, a consistent and significant neoantigen-specific CD4^+^TNFα^+^ T response, although at a low extent, was induced by αCTLA-4 alone in all treated mice. These responses further increased in mice treated with αCTLA-4/C20-IM. Treatment with αCTLA-4/CD20-ID showed a significant increase in CD4^+^TNFα^+^ but not in CD8^+^IFNγ^+^ T cells. The results suggest that the anti-tumor mechanism mediated by αCTLA-4 treatment in the IM vaccination may skew the neoantigen-specific immune response toward a CD8^+^IFNγ^+^ effector function whereas in the ID vaccination in the direction of CD4^+^TNFα^+^. Treatment with αPD1/C20-IM or αPD1/C20-ID resulted in less frequent measurable responses in two or three out of six mice. We then asked whether the neoantigen-specific immune responses were measurable in survival mice at day 258. Mice cotreated with αCTLA-4/C20-IM showed a neoantigen-specific polyfunctional CD8^+^IFN-γ^+^TNFα^+^ CD8 effector memory T cell response which was even more evident for the CD4 cells with respect to the other mice (Supplementary Fig. 6). It is tempting to speculate that the long-lasting effector memory response can contribute to the survival observed in mice treated with α αCTLA-4/C20-IM.

**Fig. 6:**
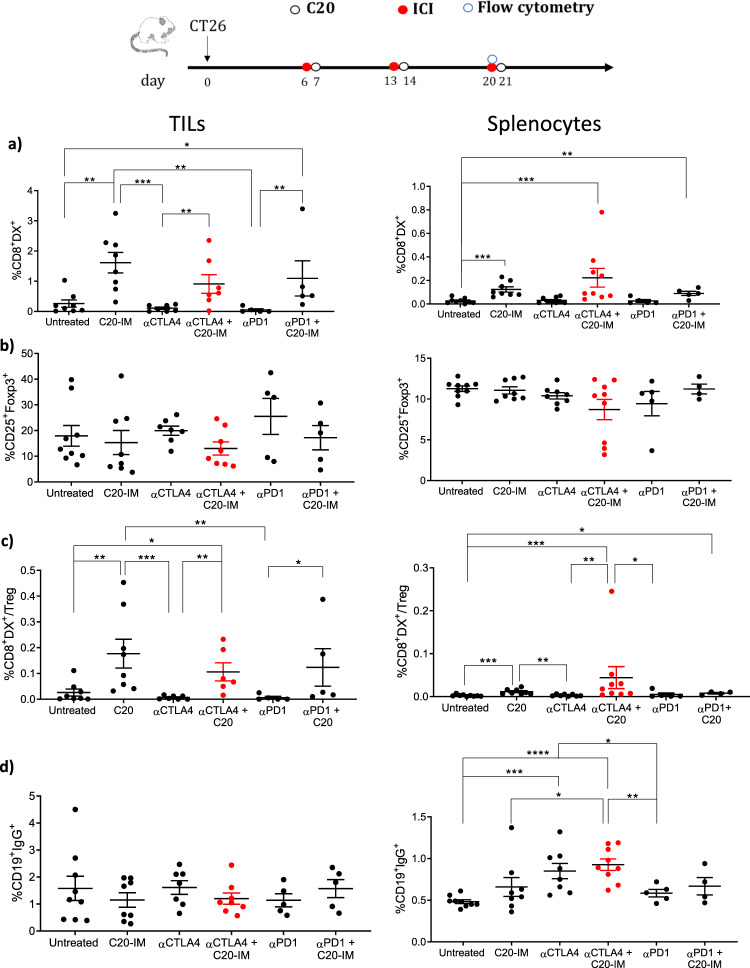
C20 and αCTLA-4 increased neoantigen-specific CD8 and switched B cells. On day twenty, nine mice per group were sacrificed and tumors and splenocytes were analyzed by FC using the gating strategy depicted in Supplementary Fig. 2. **a** Frequency of E2f8 neoantigen-specific response was measured using the specific dextramer (Dx+) gated on CD45^+^CD3^+^ CD8^+^ in live cells. **b** Treg population (CD25^+^Foxp3^+^) were gated on CD45^+^CD3^+^CD4^+^ in live cells and expressed as ratio with DX^+^ cells (**c**). **d** Switched B cells were identified as CD19^+^IgG^+^ cells and gated on CD45^+^CD3^+^ in live cells. Nine animals per group were utilized and each symbol represents an individual sample. Significance was determined using Mann–Whitney test *p < 0.05 **p < 0.01 ***p < 0.001 ****p < 0.0001.

We then looked at the intratumoral lymphocytes as compared to splenocytes. CD4^+^ and CD8^+^ T cells showed an increased frequency in the tumor but not in the spleen while nonsignificant trend was observed for CD19^+^ B cells (Supplementary Fig. 7A). In contrast, a statistically significant increase of neoantigen-specific T cells was detected in both tissues of C20-IM vaccinated mice (Fig. 6A). Analysis of granzyme b expression revealed a nonsignificant trend (Supplementary Fig. 7B). CTLA-4 is expressed by regulatory T cells and it has been shown that in mice but not in humans αCTLA-4 can reduce this cell population^39^. In this experimental setting we did not observe a reduction of the frequency of CD4^+^Foxp3^+^ Treg cells (Treg) but a significant increase of neoantigen-specific CD8/Treg ratio (Fig. 6B, C). Other factors may contribute to a strong immune response, recent evidence suggests a role of switched B cells in the antitumor activity mediated by αCTLA-4^40^. To check this, we measured the frequency of CD19^+^IgG^+^ cells. A statistically significant increase was observed in the splenocytes of mice treated with αCTLA-4 alone or with C20-IM with respect to control mice or mice vaccinated with C20-IM (Fig. 6D). This effect was specific for αCTLA-4 in the splenocytes and was not observed in mice treated with αPD1. Altogether these results further support the combined treatment of αCTLA-4/ C20-IM. Further experiments are required to correlate this observation with the enhanced antitumor effect.

## Discussion

The creation of a successful therapeutic protocol that combines ICI and NCV is currently a work in progress. The induction of multi-epitopic and multi-functional immune responses by NCV was shown to be relevant in the MC38 tumor model^15^ and other tumor models^31,41^. Here we extended this concept and explored the strength of DNA-EP platform confirming the immunogenicity of neoantigen previously used in other delivery systems or in the form of shorter coding sequencing (Tables 1 and 2). With the goal of improving the therapeutic index of αCTLA-4 and αPD1, we set experimental conditions that resulted in the expected efficacy for ICI^42^. Unlike other NCV delivery technologies showing synergetic effects with αPD1^31,43,44^, NCV delivered by DNA-EP was more effective in conjunction with αCTLA-4. In the MC38 tumor model, the reduced tumor volume correlated with an increased neoantigen-specific CD8 immune response in the splenocytes (Fig. 2C) and the peripheral blood of CT26 tumor model (Fig. 5A). This observation is coherent with the role of CTLA-4 in regulating T cell activation. It has been shown that, upon TCR ligation, CTLA-4 is upregulated and outcompetes CD28 for B7 ligand binding limiting positive costimulation by CD28^45^. An additional mechanism of action of αCTLA-4 is the upregulation of CD86 on migratory DC^46^, which in turn should favor a stronger T cell response. In line with the role played by αCTLA-4, we observed a statistically significant induction of a neoantigen-specific CD4^+^TNFα^+^ in tumor-bearing mice (Fig. 5D). This low level of induction was not observed in parallel treatment with αPD1 and was significantly boosted by C20-IM treatment further supporting the synergistic effect.

We investigated multiple therapeutic protocols and found that αCTLA-4 administration on the day before NCV treatment is the more effective schedule for established tumors. A similar protocol was originally explored in an orthotopic tumor model^27^. The mechanism of action of the cell lysate vaccine was based on the activation of NK and CD4 T cells. Here we applied this schedule with NCV and showed that effector neoantigen-specific immune responses were induced in the periphery as well as in the tumor. Differently from C20-ID, the C20-IM vaccine protocol resulted in a CD8^+^IFN^+^ effector memory (Fig. 3A and Supplementary Fig. 6A), and in the therapeutic treatment with αCTLA-4/C20-IM resulted in long-lasting regression of the CT26 tumor model (Fig. 5B). Although the neoantigen-specific immune response in the periphery of tumor-bearing mice was not a clear biomarker of the antitumor response it suggests that the quality of the responses rather than the site of vaccination, ID vs. IM, is an important determinant. Our preliminary data identify some differences in the immune responses between C20-ID and C20-IM, however, we cannot exclude that other electrical conditions may favor neoantigen-specific CD8^+^IFN^+^ T cells immune response in the ID protocol^47,48^. The lack of antitumor activity of NCV with αPD1 in this therapeutic protocol is in line with previous evidence showing that treatment with αPD1 before vaccination had a negative impact on tumor growth^49^. The mechanism seems to be based on the induction of dysfunctional PD1^+^CD38^hi^CD8^+^ T cells in sub optimally primed CD8 T cell conditions induced by tumors. Previous evidence in mice and humans showed that nonreversible chromatin modification is induced in non-responding patients and at late-stage tumor growth in preclinical animal models with the CD8 cells expressing CD38 and CD101^50^. The possibility that priming of T cell response is requested before αPD1 is likely to occur in our experimental conditions. The data from Verma and colleagues^49^ also suggest that in the absence of a good T cell priming, i.e. by NCV, the therapeutic potential of T cells targeting neoantigens may be irreversible committed to a nonresponsive phenotype.

The mechanism of action of NCV and αCTLA-4 does not involve only the strengthening of peripheral CD4 and CD8 neoantigen-specific immune response (Fig. 5) but correlates with switched B cells in the spleen (Fig. 6). Importantly, our findings are specific for αCTLA-4. Thibult et al. described αPD1 antibodies directly activating peripheral B cells that had high PD1 expression in a T cell-independent mechanism^51^. In addition, they did not observe any impact of αPD1 on the production of class-switched antibodies. Others have shown that PD1-high B cells functionally suppress T cell activity^52^. In the combined treatment with αCTLA-4, we observed a slight but not significant decrease of neoantigen-specific immune responses associated with 100% protection in the CT26 tumor model. The increased frequency of antibody switched B cells observed in αCTLA-4 treated mice is in line with previous evidence in other tumors using αCTLA-4 alone or with αPD1. An increased B cell activation (CD19^+^ MHC-II^+^ CD80/CD86^+^), as well as an increased antibody titer against the cancer cells, was observed in breast cancer tumor models^40^. In the CT26 tumor model, we could observe a statistically significant increase of switched B cells only with αCTLA-4 and not with αPD1 in the spleen where they may serve *as* antigen-presenting cells boosting the T cell response.

The therapeutic protocols utilized were well tolerated including the treatment with dual ICI, which are associated with relevant side-effects in the clinic. Although mouse tumor models are not commonly utilized for toxicology studies, we did not observe any sign of discomfort during the 3 weeks treatment in C57/B6 and Balb/C mice or in the long follow-up in αCTLA-4 and C20 treated mice. It is tempting to translate this in the clinic where a therapy based on DNA-EP and αCTLA-4 could be more appealing than αCTLA-4/ αPD1 due to possible reduced side effects. In conclusion, our data support the use of NCV delivered by DNA-EP with αCTLA-4 suggesting new protocols for clinical testing.

## Methods

### Vaccines, cell lines, and mice

Vaccines specific for each tumor cell line were generated as a fusion protein with the TPA leader sequence upon codon optimization. As for the MC38-specific vaccine, named M8, the neoantigens listed in Table 1 were inserted one after the other without linkers; M2h was produced for the MC38 cell line by inserting at the C terminus of M2 vaccine^15^ the CD4 helper-epitopes FNNFTVSFWLRVPKVSASHLE, AKFVAAWTLKAAAW, and AWLEAQEEEEVGF^53^ were connected one after the other using furin linkers C20 plasmid vaccine specific for the CT26 cells was generated with the neoantigen listed in Supplementary Table 1 without linkers. MC38 and CT26 colon carcinoma cell lines were purchased from Kerafast and ATCC respectively. Master and working cell banks were generated upon receipt, with their third and fourth passages being used for all tumor challenge experiments. Cells were mycoplasma-free as per internal regular controls. In vivo luciferase expression was measured using Xenogen IVIS300 as previously described^54^ Female 6–8 weeks old C57BL/6 and Balb/c mice (Envigo) were housed in the Plaisant animal house according to national legislation and kept in standard conditions according to Takis ethical committee approval. All the in vivo experimental procedures were approved by the local animal ethics council. The ethical committee of the Italian Ministry of Health approved this research, authorization # 586/2019-PR. Mouse experiments were conducted with a variable number of animals as described in the figure legends.

### Immunization schedule

In total, 10 μg of plasmid DNA, were injected in a 50 μL volume into the tibialis muscle followed by electroporation, as previously described^15^. For ID administration, C20 were injected in a 30 μl volume intradermally in the left flank and followed by EP (40 V, 3 pulses, 100 ms length, 120 ms pause) as previously described^55^. 200 μg of anti-PD1 or anti-CTLA4 were injected in a 200 μL volume in mice peritoneum. Tumor challenge was performed by injecting 3 × 10^5^ MC38 cells on C57Bl/6 mice or 1 × 10^6^ CT26 on Balb/c mice subcutaneously (s.c.), in a 100 μL PBS volume in the right flank of the mice. Tumor growth was monitored twice a week using an electronic caliper.

### In vivo depletion of CD4 and CD8

CD4 and CD8 were depleted in vivo using anti-CD4 (clone GK1.5, Bioxcell) and anti-CD8 (clone 53–6.7, Bioxcell) antibodies. 200 μg of antibody were injected in a 200 μL volume in mice peritoneum, twice a week for three weeks.

### Immune responses

The neoantigen-specific immune responses were determined in splenocytes, PBMCs, and tumors of the mice by using intracellular cytokine staining (ICS) performed by flow cytometry (FC), as previously described^15^. Briefly, PBMCs or splenocytes were incubated for 10 min at room temperature in ACK (Ammonium-Chloride- Potassium) Lysing Buffer (Life Technologies) and then washed in RPMI-1640 medium (Gibco-BRL) supplemented with 10% fetal bovine serum (FBS, Life Technologies). Tumors harvested from the mice were mechanically dissociated using a scalpel and incubated for 40 min at 37 °C with a cocktail of enzymes from the Miltenyi Tumor Dissociation kit in RPMI-1640. Then, tumors were passed through a 70 μm Cell Strainer and washed in RPMI-1640 medium with 10% FBS. For FC analysis of IFNγ and TNFα producing cells, 1 × 10^6^ PBMCs or splenocytes were cultured in 96-well plates and stimulated for 12–16 h in 10% FBS-supplemented RPMI-1640 with the pool of MC38 or CT26 neoantigen peptides at the final concentration of 5 μg/ml, DMSO or PMA-ionomycin, as previously described^15^. For FC analysis, dead cells were excluded by using Fixable Viability Stain 575 V (BD, cat. 565694). Cells were incubated with the anti-Fcγ receptor (2.4G2) followed by staining with the following antibodies according to different panels: CD3 (142-2C11) AF488 diluted 1:300 (Thermo Fisher Scientific, cat. 53-0031-82), FOXP3 (FJK-16s) PE diluted 1:100 (Thermo Fisher Scientific, cat. 12-5773-82), CD4 (RM4-5) PerCP-eFluor 710 diluted 1:400 (Thermo Fisher Scientific, cat. 46-0042-82), CD19 (1D3) PC7 diluted 1:300 (Thermo Fisher Scientific, cat. 46-0042-82), CD8 (53-6.7) APC-eFluor 780 diluted 1:300 Thermo Fisher Scientific, cat. 47-0081-82), CD45 (30-F11) eFluor 450 diluted 1:500 (Thermo Fisher Scientific, cat. 48-0451-82), IgG1 (A85-1) BV605 diluted 1:200 (BD, cat. 563285), IgG2a/b BV605 (BD, cat. 744294), CD25 (PC61.5) SB780 diluted 1:100 (Thermo Fisher Scientific, cat. 78-0251-82), IFNγ (XMG1.2) PE diluted 1:150 (Thermo Fisher Scientific, cat. 12-7311-82), TNFα (MP6-XT22) PC7 diluted 1:400 (Thermo Fisher Scientific, cat. 25-7321-82) and MHC Dextramer (H-2Dd/SGPSYATYL) PE (Immudex) (1:25 dilution) specific for the E2f8 neoantigen. The stained samples were acquired through a CytoFLEX flow cytometer (Beckman Coulter), and the data were analyzed using CytExpert software (Beckman Coulter). Cytokine expression in the presence of only DMSO (with no peptides) was considered background and subtracted from the values measured with stimuli.

### IFN-γ ELIspot

The assay was performed on splenocytes from vaccinated and control mice according to the manufacturer’s instructions (Mouse IFN-gamma ELISpotBASIC ALP, Mabtech). Briefly, standard 96-well plates (Millipore) were coated with anti-mouse IFNγ antibody diluted to 15 µg/ml in sterile PBS and blocked with RPMI-1640 medium with 10% FBS. Splenocytes were plated at 4 × 10^5^ and 2 × 10^5^ cells/well, in duplicate, with the pool of MC38 and CT26 neoantigen at the final concentration of 1 μg/ml. After overnight stimulation at 37 °C, plates were washed and incubated with biotinylated anti-mouse IFNγ antibody, washed, and incubated for 1 h at room temperature with streptavidin-AP conjugated antibody. After washing, 50 μl/well of the substrate (NBT/BCIP-1 step solution, Pierce) was added to measure spot development. After incubation of about 30 min at RT, the plates were thoroughly washed with distilled water to stop the reaction. Plates were allowed to air-dry completely, and spots were counted using an automated ELISPOT reader (Aelvis ELIspot reader, A.EL.VIS Gmbh).

### RT-PCR

RNA was extracted from 30 mg of frozen tumor tissues according to the manufacturer’s instructions (RNeasy kit, QIAGEN). To smash and homogenize tissues, a TissueLyser LT (QIAGEN) was used and operated at 50 Hz for 2 min. RNA extracted was retro-transcribed to cDNA using a high capacity cDNA Reverse Transcription Kit (Applied Biosystems) according to the manufacturer’s instructions. Real-time (RT)-PCR was performed using TaqMan™ Gene Expression Master Mix (Applied Biosystem) and commercial TaqMan probes Gzmb genes (Applied Biosystem). Gene expression was normalized to 18S and expressed using the 2^−Δct^ method.

### Luminex

Circulating cytokines and chemokines were measured by Luminex multiparameter assay. IL10, IL1b, IL2, IL4 IFNγ, TNFα, IL17, IL7, IL6, GM-CSF, MIG (CXCL9), IP10 (CXCL10), MIP1α (CCL3), and MCP1 (CCL2) were analyzed according to the manufacturer’s instruction.

### Statistical analysis

Log-rank (Mantel–Cox) and Mann–Whitney were utilized where indicated. All analyses were performed in GraphPad Prism 8.0.2.

### Reporting summary

Further information on research design is available in the Nature Research Reporting Summary linked to this article.

## Supplementary information

Supplementary Figures

Reporting Summary

## Data Availability

The data not described in the supplementary figures, including raw data and sequences, are available from the corresponding authors upon reasonable request. M8 and C20 plasmid were deposited in Addgene data base (#80536).
