# Supplementary material for: Neoantigen cancer vaccine augments anti-CTLA-4 efficacy
Source: NPJ Vaccines. 2022 Feb 2;7:15. doi: 10.1038/s41541-022-00433-9 (PMC8810847; doi:10.1038/s41541-022-00433-9)
Supplement: Supplementary file 1 — Supplementary Figures [file 41541_2022_433_MOESM1_ESM.pdf]

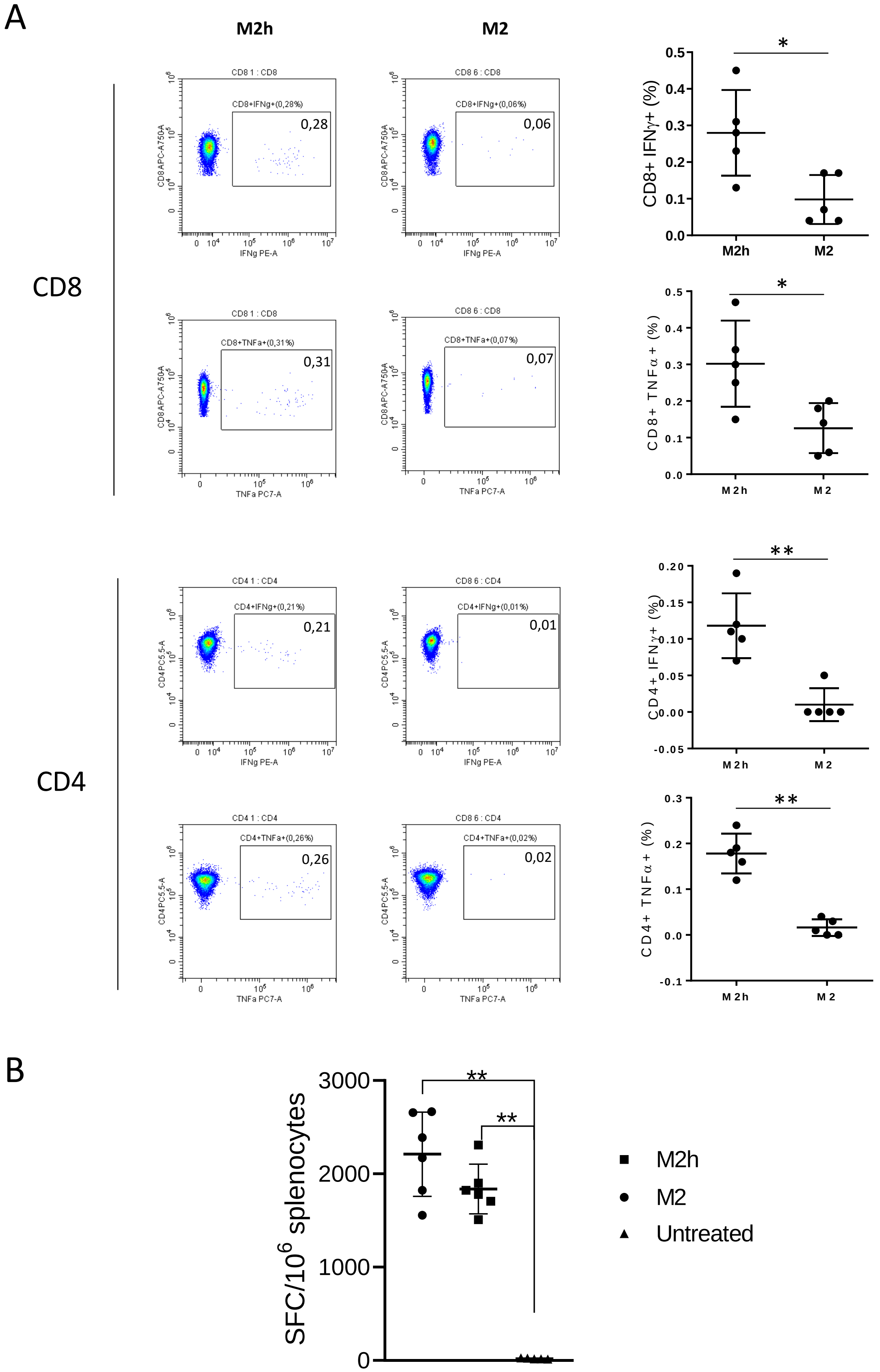

Sup. Fig 1. Immune response induced by M2 or M2h vaccine. Analysis of immune responses measured in the spleen of C57/b6 mice vaccinated with M2 or M2h at day 0, 21 and 42 and immune responses analyzed one week after the last vaccination. **A)** Upper panel shows FC analysis upon stimulation with neoantigen peptides (CD8), lower panel with helper CD4 peptides (CD4). **B)** IFN-γ ELISpot assay with splenocytes restimulated with neoantigen peptides. Significance was determined using Mann-Whitney tests (*p< 0,05 **p< 0.01)

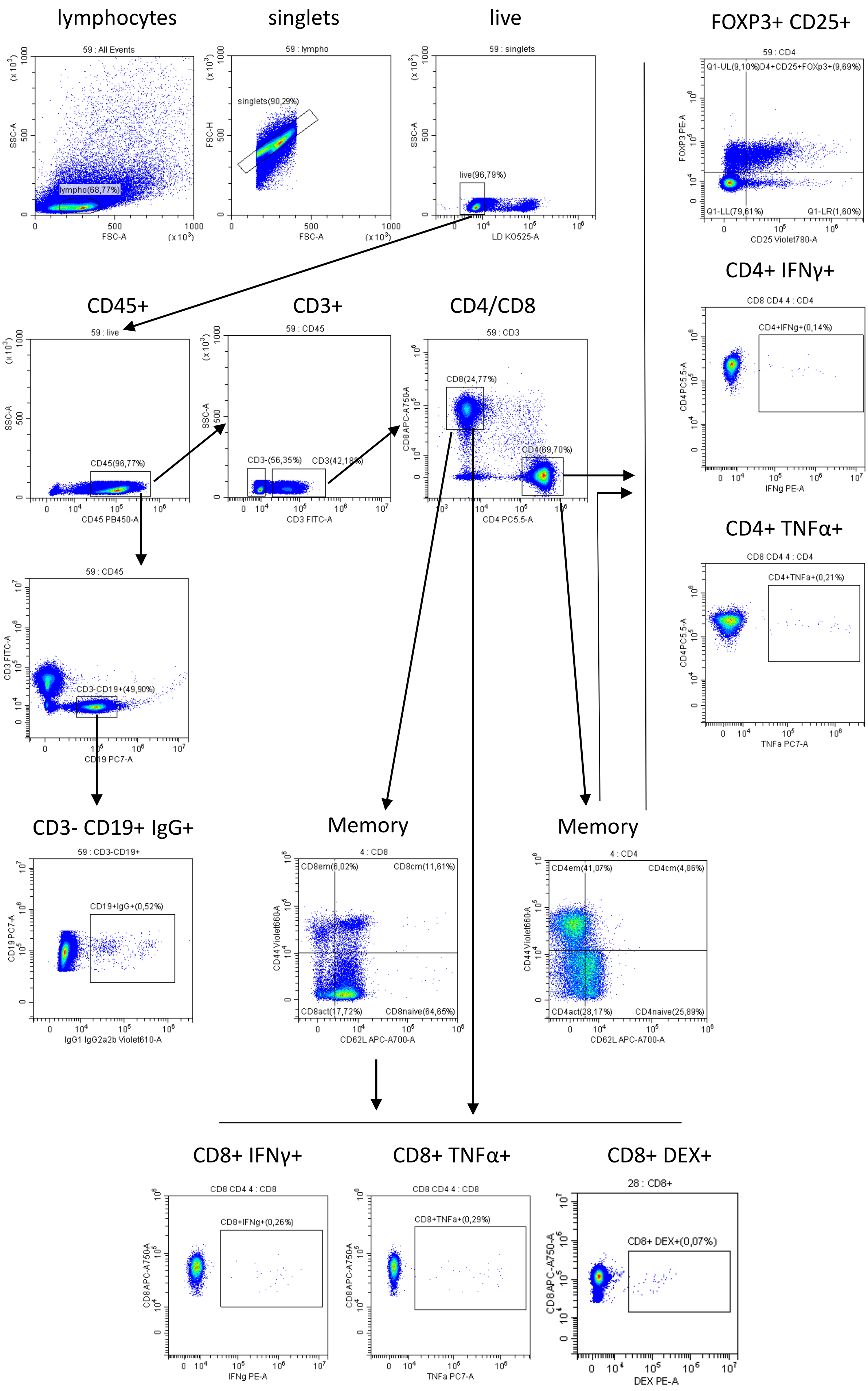

Sup. Fig.2 Gating strategy used in Fig. 2, Fig. 3, and Fig. 6. In all but the Fig.6 where dextramers were used, the cells were stimulated with the neoantigen peptide pool.

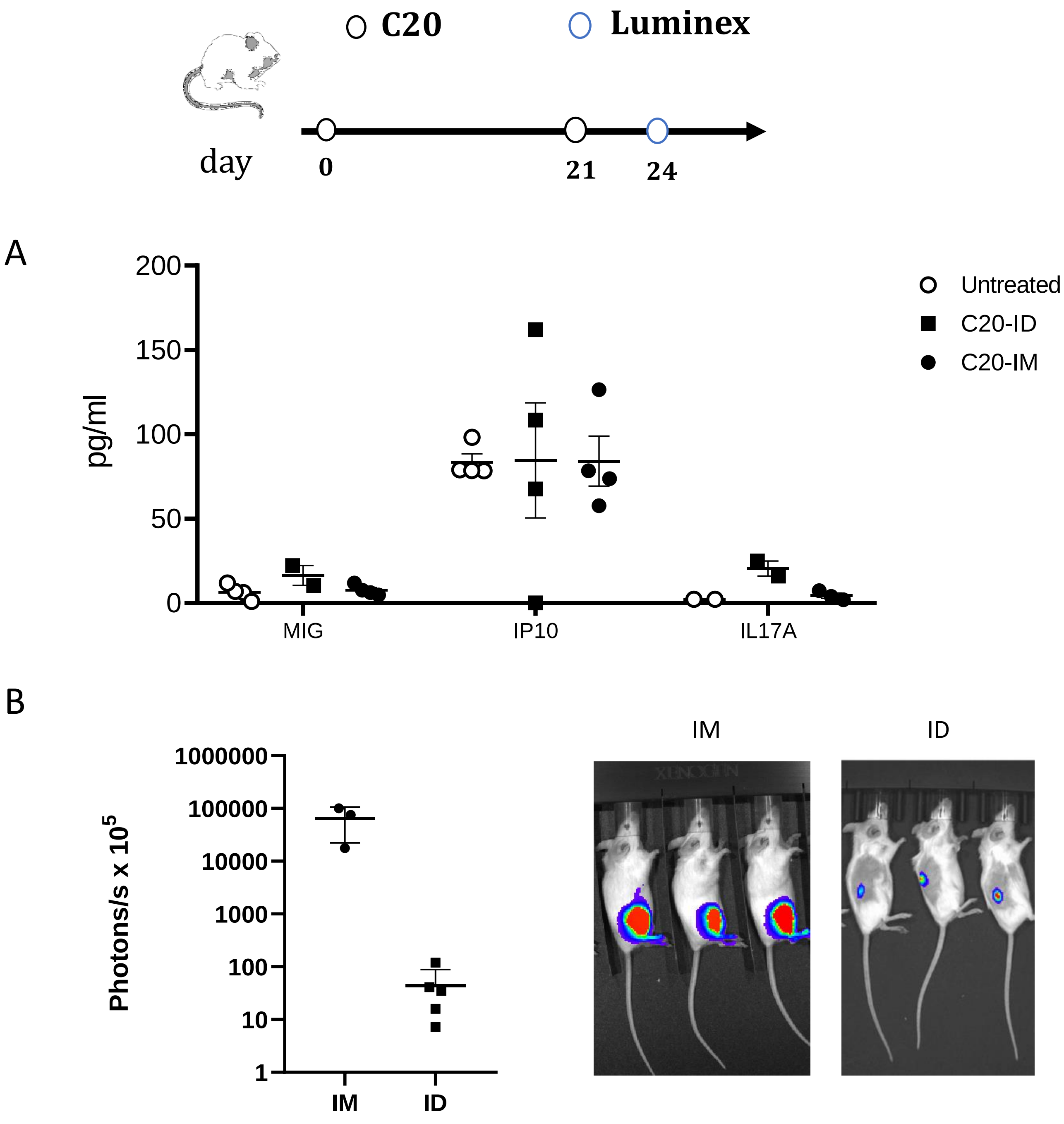

Sup. Fig. 3. A. Circulating cytokines and chemokines upon NCV. Analysis of circulating cytokines and chemokines measured in the serum of mice vaccinated with C20-ID or C20-IM using the protocol depicted in Fig.3 up to day 24 when mice were bled (3 days after the second vaccination). Selected markers measured by Luminex assay are reported. **B Luciferase expression in ID and IM protocol**. Mice were injected with luciferase expressing plasmid and subjected to the ID or IM protocol used for the vaccination study. In the graph are reported the data as photons per second on the right representative imagines

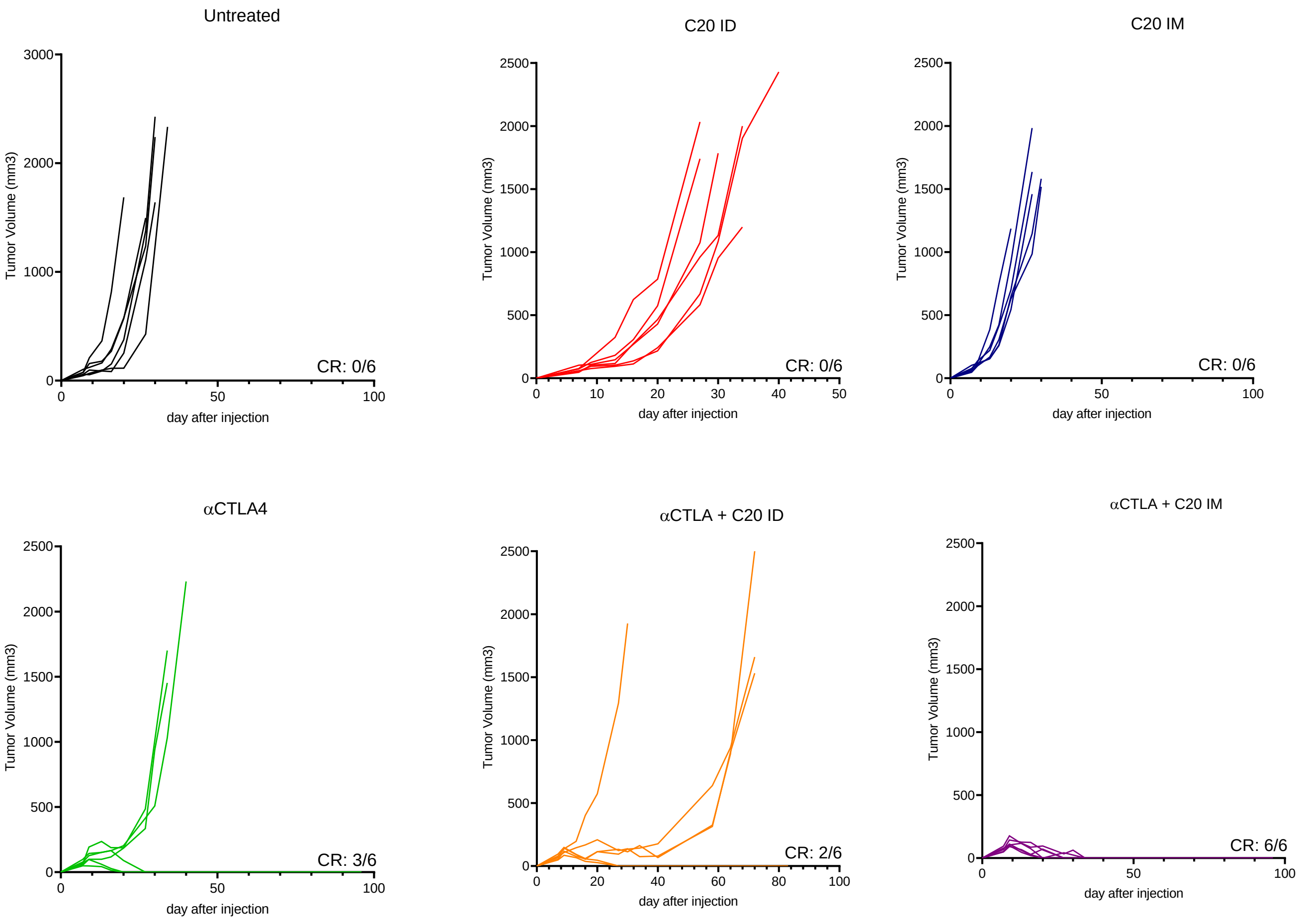

Sup. Fig 4 Tumor volumes of single mice. Mice were treated as depicted in Fig.4 B, single tumor volumes were measured over time. Ratio depicts frequency of mice with complete response (CR)

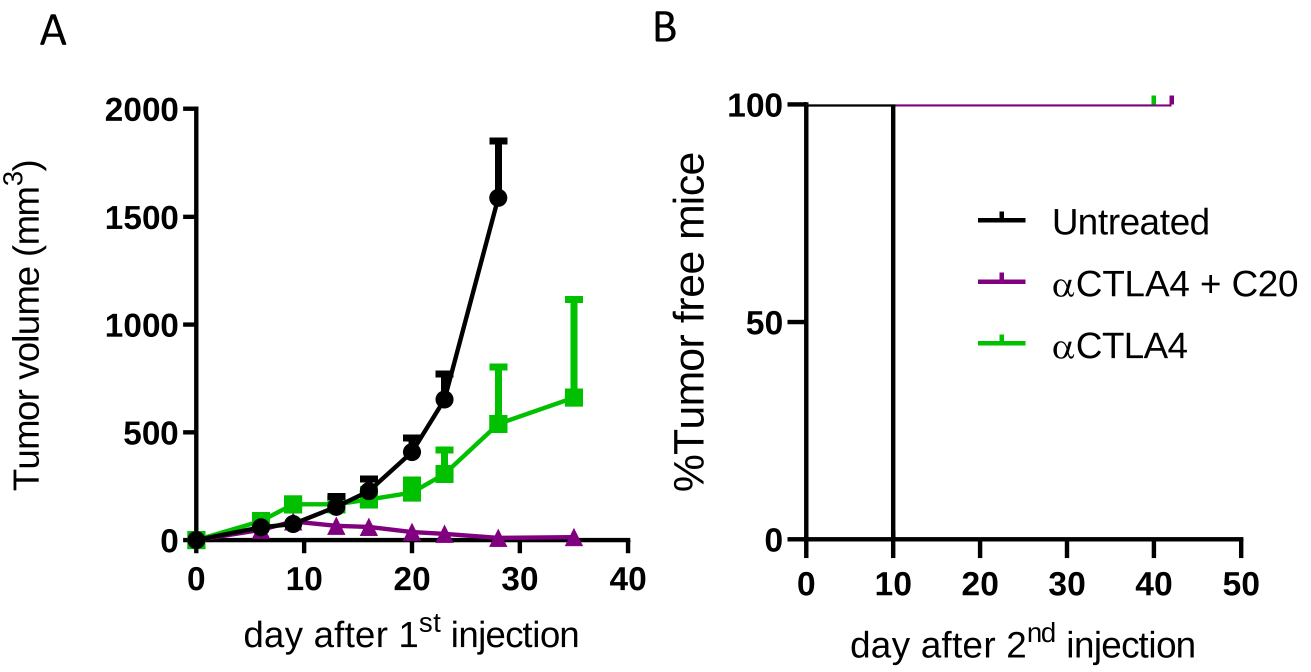

Sup. Fig 5 Tumor re-challenge in CT26 survivor mice. Following the experimental scheme depicted in Fig.4 B mice were treated with αCTLA-4, αCTLA-4 + C20 or left untreated. **A** First tumor challenge. **B** Second tumor challenge. At day 70 mice surviving the first tumor challenge (αCTLA-4 n=3 and αCTLA4 + C20 n=6) were injected in the opposite flank with CT26 tumor cells. Untreated were mice not previously treated with CT26 tumor cells. Six mice per group were utilized.

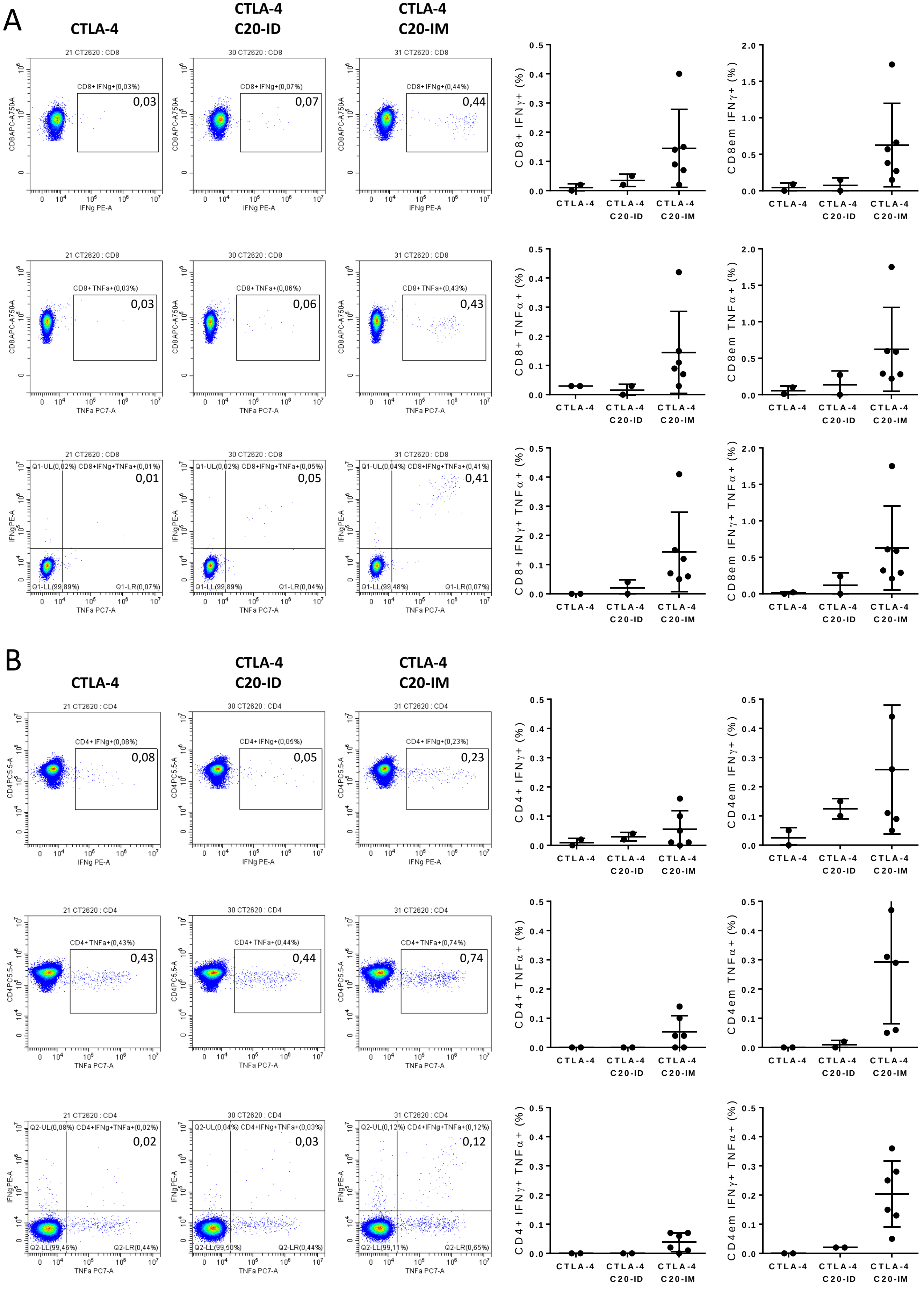

Sup. Fig . 6 Memory immune response in survivor mice. Analysis of neoantigen-specific immune responses measured in the spleen of mice Balb/c mice vaccinated as described in the scheme of Fig. 4B and sacrificed at day 258. **A)** FC analysis of CD8. **B)** FC analysis of CD4.

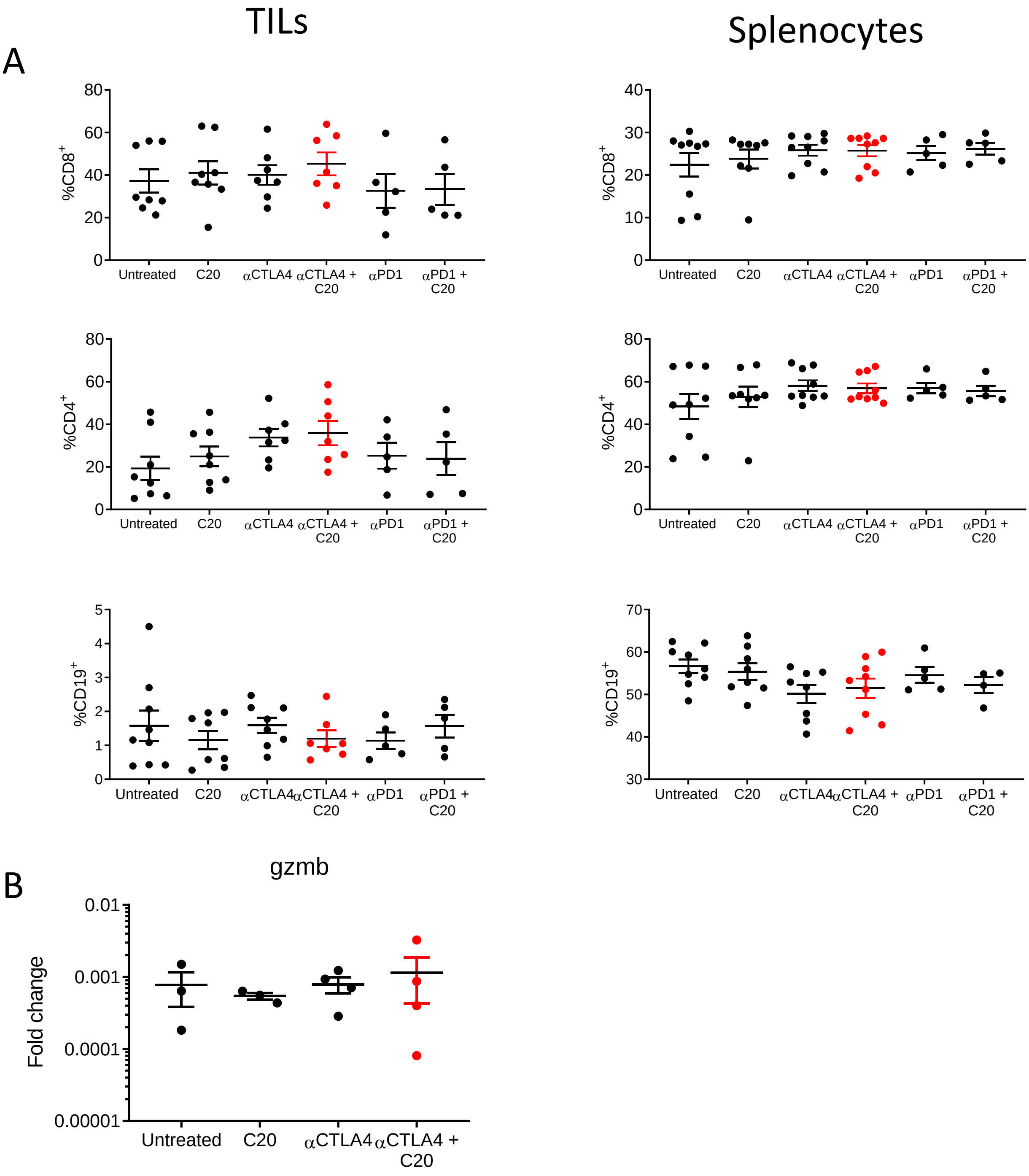

Sup. Fig . 7 On day 20, 9 mice per group were sacrificed and tumors and splenocytes were collected. **A**) Tumors and splenocytes were analyzed by FC . Frequency of CD8+ $CD4^+$ populations were gated on $CD45^+$ $CD3^+$ in live cells. $CD19^+$ B cell were gated on $CD45^+$ $CD3^-$ in live cells. Mann-Whitney tests were conducted. **B**) Tumor expression of gzmb gene assessed by RT-PCR.

Supp table 1: C20 vaccine expressing CT26 neoantigens

	WT	Neoantigen
**Symbol**	**Seq**	**Seq**
E2f8	VILPQAPSGPSYA**I**YLQPAQAQMLTPP	VILPQAPSGPSYA**T**YLQPAQAQMLTPP
Aldh1	LHSGQNHLKEMAI**P**VLEARACAAAGQS	LHSGQNHLKEMAI**S**VLEARACAAAGQS
Slc4a3	PLLPFYPPDEALE**T**GLELNSSALPPTE	PLLPFYPPDEALE**I**GLELNSSALPPTE
Nphp3	AGTQCEYWASRAL**G**SEHSIGSMIQLPQ	AGTQCEYWASRAL**D**SEHSIGSMIQLPQ
Tdg	AAYKGHHYPGPGN**H**FWKCLFMSGLSEV	AAYKGHHYPGPGN**Y**FWKCLFMSGLSEV
Ubqlnl	DTLSAMSNPRAMQ**A**LLQIQQGLQTLAT	DTLSAMSNPRAMQ**V**LLQIQQGLQTLAT
Slc20a1	DKPLRRNNSYTSY**T**MAICGMPLDSFRA	DKPLRRNNSYTSY**I**MAICGMPLDSFRA
Dhx35	EVIQTSKYYMRDV**T**AIESAWLLELAPH	EVIQTSKYYMRDV**I**AIESAWLLELAPH
Als2	GYISRVTAGKDSY**T**ALVDKNIMGYIAS	GYISRVTAGKDSY**I**ALVDKNIMGYIAS
Agxt2l2	EHIHRAGGLFVAD**E**IQVGFGRIGKHFW	EHIHRAGGLFVAD**A**IQVGFGRIGKHFW
Tmem87	QAIVRGCSMPGPW**G**SGRLLVSRRWSVE	QAIVRGCSMPGPW**R**SGRLLVSRRWSVE
Ppp6r1	DGQLELLAQGALD**D**ALSSMGALHALRP	DGQLELLAQGALD**N**ALSSMGALHALRP
Deptor	SHDSRKSTSFMSV**S**PSKEIKIVSAVRR	SHDSRKSTSFMSV**N**PSKEIKIVSAVRR
Nap1	HTPSSYIETLPKA**V**KRRINALKQLQVR	HTPSSYIETLPKA**I**KRRINALKQLQVR
Cxcr7	MKAFIFKYSAKTG**F**TKLIDASRVSETE	MKAFIFKYSAKTG**L**TKLIDASRVSETE
Dkk2	EGDPCLRSSDCID**G**FCCARHFWTKICK	EGDPCLRSSDCID**E**FCCARHFWTKICK
Trip1	WKGGPVKIDPLAL**V**QAIERYLVVRGYG	WKGGPVKIDPLAL**M**QAIERYLVVRGYG
Steap2	VTSIPSVSNALNW**R**EFSFIQSTLGYVA	VTSIPSVSNALNW**K**EFSFIQSTLGYVA
Gpc1	YRGANLHLEETLA**E**FWARLLERLFKQL	YRGANLHLEETLA**G**FWARLLERLFKQL
Usp26	KTTLSHTQDSSQS**S**QSSSDSSKSSRCS	KTTLSHTQDSSQS**L**QSSSDSSKSSRCS
